# COVID-19 associated acute transplant failure after AB0-incompatible living donor kidney transplantation – a case report

**DOI:** 10.1186/s12882-023-03070-z

**Published:** 2023-01-24

**Authors:** Kristina Boss, Margarethe Konik, Jan Hinrich Bräsen, Jessica Schmitz, Christiane Jürgens, Andreas Kribben, Oliver Witzke, Sebastian Dolff, Anja Gäckler

**Affiliations:** 1 Department of Nephrology, University Hospital Essen, University Duisburg-Essen, Hufelandstr. 55, 45147 Essen, Germany; 2 Department of Infectious Diseases, University Hospital Essen, University Duisburg-Essen, Essen, Germany; 3 Nephropathology Unit, Institute of Pathology, Hannover Medical School, Hannover, Germany

**Keywords:** AB0-incompatible, Kidney transplantation, COVID-19, Rejection, Case report

## Abstract

**Introduction:**

Immunosuppressive therapy is associated with an increased risk of severe courses of SARS-CoV-2 infection, with frequently delayed viral clearance. We report a case of an acute kidney transplant failure in persistent SARS-CoV-2 infection in a patient with absolute B-cell depletion after administration of rituximab for AB0-incompatible living donor kidney transplantation.

**Case presentation:**

A 34-year-old unvaccinated patient is diagnosed with SARS-CoV-2 infection four months after kidney transplantation. With only mild symptoms and an estimated glomerular filtration rate (eGFR) of 44 ml/min/1.73 m^2^, therapy with molnupiravir was initially given. Within the next eight weeks, transplant biopsies were performed for acute graft failure. These showed acute T-cell rejection with severe acute tubular epithelial damage with only mild interstitial fibrosis and tubular atrophy (BANFF cat. 4 IB), and borderline rejection (BANFF cat. 3). A therapy with prednisolone and intravenous immunoglobulins was performed twice. With unchanged graft failure, the third biopsy also formally showed BANFF cat. 4 IB. However, fluorescence in situ hybridization detected SARS-CoV-2 viruses in large portions of the distal tubules. After nine weeks of persistent COVID-19 disease neither anti-SARS-CoV-2 IgG nor a SARS-CoV-2-specific cellular immune response could be detected, leading to the administration of sotrovimab and remdesivir. Among them, SARS-CoV-2 clearance, detection of IgG, and improvement of graft function were achieved.

**Conclusion:**

Lack of viral clearance can lead to complications of SARS-CoV-2 infection with atypical manifestations. In kidney transplant patients, before initiating therapy, the differential diagnoses of “rejection” and “virus infection” should be weighed against each other in an interdisciplinary team of nephrologists, infectious diseases specialists and pathologists.

## Introduction

Covid-19 produces high burden of inflammation. In transplant recipients this effect may be alleviated by immunosuppressive drugs [1]. On the other hand, immunosuppressive therapy is associated with an increased risk of severe courses of SARS-CoV-2 infection, with frequently delayed viral clearance and the COVID-19 related mortality rate is higher in kidney transplant recipients than in nontransplant patients [4]. We report a case of an acute kidney transplant failure in persistent SARS-CoV-2 infection in a patient with absolute B-cell depletion after administration of rituximab for AB0-incompatible living donor kidney transplantation.

### Case presentation

A 34-year-old unvaccinated man is diagnosed with SARS-CoV-2 infection by a naso-pharyngeal swab and reverse transcription polymerase chain reaction (PCR) assay four months after transplantation. The immunosuppressive therapy consisted of tacrolimus, mycophenolate mofetil and prednisone. Hospitalisation for remdesivir therapy was refused by the patient. A SARS-CoV-2 neutralizing antibody was not available at this time (Jan 2022), so with an eGFR of 44 ml/min/1.73 m^2^ and only mild symptoms, a therapy with molnupiravir was initially given. The patient did not report any drug-related adverse reactions. There were no thrombocytopenia or elevated transaminases.

In the following weeks there was a progressive deterioration in transplant function, so that the patient was finally admitted to hospital four weeks after onset of infection. Blood analysis showed leukopenia and an absolute B-cell depletion. The first transplant biopsy demonstrated an acute T-cell rejection with severe acute tubular epithelial damage with only mild interstitial fibrosis and tubular atrophy (BANFF cat. 4 IB). The patient received prednisolone intravenously over three days with a cumulative dose of 1 g.

As the transplant function did not improve, a second biopsy was performed, which demonstrated a borderline rejection (BANFF cat. 3). This was followed by a second prednisolone therapy as well as the administration of intravenous immunoglobulins (iVIG) with a cumulative dose of 60 g over three days. Tacrolimus trough levels were measured at short intervals and were within the therapeutic range of 5–7 ng/ml. There was no evidence for donor specific binding HLA IgG antibodies.

**Fig. 1:**
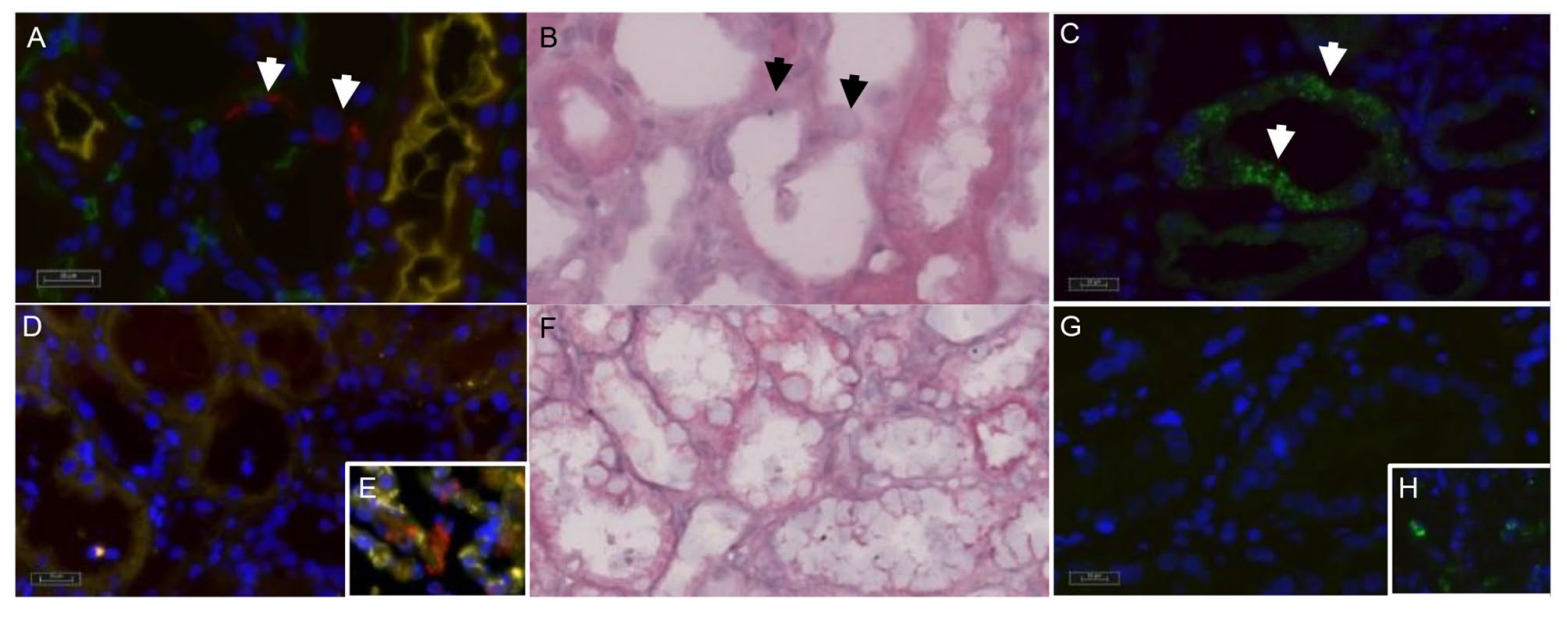
Histopathology of renal biopsies: A, B and C: Third biopsy shows SARS-CoV2 positive FISH (A, SARS-CoV2 RNA signals in red (arrowheads), with combined immunofluorescence for endothelial cells in green (CD34), proximal tubules in yellow (CD10), B: identical section stained H&E, C: Nucleocapsid immunofluorescence in green. D, F, G: Seven weeks later, control biopsy does not show positive signals for SARS-CoV2-RNA (D), F: identical section stained H&E, G: nucleocapsid immunofluorescence. E: positive control (Covid-19 pneumonia) for SARS-CoV-2-RNA, H: same sample with positive nucleocapsid immunofluorescence. Scale 20 μm (A and D), Scale 10 μm (C and G)

Approximately eight weeks after the onset of infection, the patient developed a COVID-19 pneumonia with bacterial superinfection, which was treated anti-infectively with tazobactam/piperacillin and clarithromycin and immunomodulatory with dexamethasone (6 mg/d for 7 days) and renewed iVIG. Several microbiologic tests have been performed without identification of a certain pathogen. So, blood and urine analyses, fungal diagnostics in blood and bronchoalveolar lavage and mycoplasma tests remained negative. With unchanged highly restricted graft function, a third biopsy showed formally acute T-cell rejection, BANFF cat. 4 IB. However, fluorescent in situ hybridization (FISH), using the XRNA SARS-CoV-2 RNA FISH probe (MetaSystems Probes, Altlußheim, Germany) and immunofluorescent stain using a polyclonal rabbit antibody directed against SARS-CoV-2 nucleocapsid protein (Novus Biologicals, Centennial, CO, USA) were able to detect SARS-CoV-2 viruses and nucleocapsid protein in numerous distal tubules [Fig. 1 A, B, C]. Spike protein was detectable in lung but not in renal biopsies with polyclonal rabbit antibody (ProSci, Poway, CA, USA; data not shown). Unfortunately, it was not possible to detect SARS-CoV-2 in urine. Clinico-pathological discussion of all patient data led to the conclusion, that the immune cell infiltration should be interpreted as caused by infection rather than rejection.

After nine weeks of continuous infection with a cycle threshold between 22 and 28, neither anti-SARS-CoV-2 IgG nor a ELiSpot specific cellular immune response could be detected, leading to the administration of remdesivir and sotrovimab (the latter was available in the meantime). Due to the patients eGFR of 16 ml/min1.73 m^2^ this therapy was an off-label treatment. Among these, a SARS-CoV-2 clearance, the detection of anti-SARS-CoV-2 IgG, a positive SARS-CoV2 ELiSpot reaction and an improvement in graft function were achieved. The patient was discharged from hospital after 12 days.

**Fig. 2:**
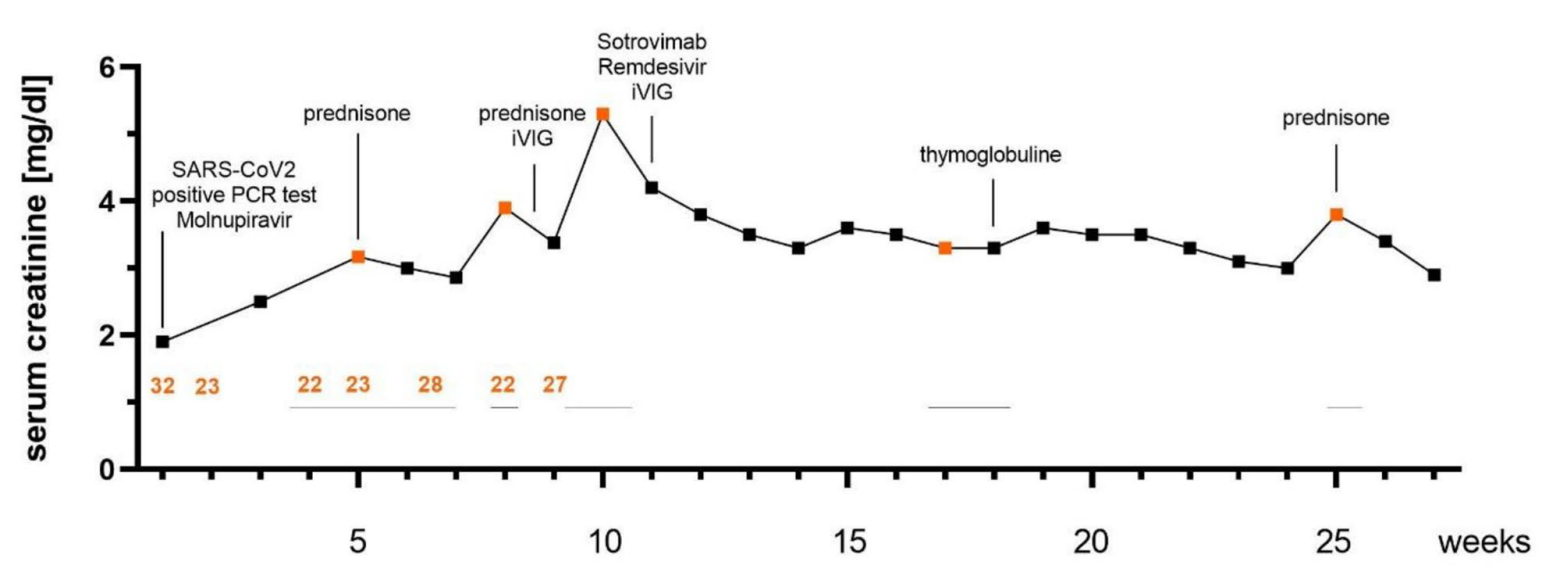
Course of serum creatinine in the context of biopsies and treatments performed Figure shows course of serum creatinine in the context of the biopsies and treatments performed, dots in orange mark dates of graft biopsies, numbers in orange mark SARS-CoV2 PCR ct values, black horizontal lines mark hospitalization periods; iVIG intravenous immunoglobulins

In the outpatient follow-up, serum creatinine (SCr) stagnated in the range of 3.5 mg/dl, so the patient was hospitalised again. The fourth biopsy (performed 19 weeks after first SARS-CoV2 positive PCR test) demonstrated again an acute T-cell rejection (BANFF cat. 4 IA). A nasopharyngeal swab yielded no evidence of SARS-CoV2 re-infection, a second FISH and immunofluorescence stains showed neither viruses nor nucleocapsid protein in the tubules and there were no viruses detectable in the urine (Fig. 1). Therefore, a therapy with thymoglobulin (300 mg cumulative) followed by renewed iVIG administration was performed. Graft function slowly improved to a SCr of 3.0 mg/dl. A control biopsy four weeks after thymoglobulin administration showed again a borderline rejection and a chronic T-cell rejection BANFF (cat. 3 + 4 IA), so that a third prednisolone shot (1 g cumulative) was given, tacrolimus trough levels were increased to 7–10 ng/ml. Additionally, 80% interstitial fibrosis and tubules atrophy (IFTA) was reported. To prove, whether this is representative for the whole graft, an ultrasound examination using contrast media was performed, showing adequate perfusion in all parts of the graft with resistance indices of 0.68–0.74. Therefore, we considered this histological finding to be most likely a sampling error. In the following days, SCr dropped to 2.9 mg/dl and remained stable in this range. An overview of the course of SCr, biopsies and treatments performed is shown in Fig. 2.

## Discussion

Patients after renal transplantation are at higher risk for a severe course of COVID-19, but even in milder courses of disease, virus clearance in particular is delayed and anti-SARS-CoV2 IgG build-up is often inadequate [2–4]. In the case reported here, virus clearance could only be achieved 9 weeks after the onset of infection and after multiple antiviral therapies.

There is growing knowledge about antiviral treatments, like molnupiravir and remdesivir, in the setting of outpatient care of immunosuppressed patients [5–7]. The data available to date show that such therapies are safe and usually well tolerated. Nevertheless, the case presented here shows that under particularly pronounced immunosuppression (here: absolute B-cell depletion following rituximab), a single antiviral therapy may not be sufficient.

In addition, some autopsy studies have also detected SARS-CoV2 in the kidney and a COVID-19 disease is associated with increased risk of acute kidney injury [8, 9]. Not only SARS-CoV2, but also other viral infections, such as BK virus, can alter immunologic homeostasis in kidney transplant patients and lead to allograft rejection. ABO incompatible kidney transplants, as described here, are at higher risk for rejection [10, 11]. The multiple interactions between infection and rejection may therefore complicate accurate diagnosis [12]. Further, both rejection and infection may alter inflammation in a patient [1]. To our knowledge, this is the first case report about the detection of SARS-CoV2 in the tubule’s cells of a kidney transplant. It reports the histopathological challenges in differential diagnosis. Even though the probe has not yet been approved for routine diagnostics, there was clear evidence of SARS-CoV2 in the FISH analysis. We assumed replication in the kidney based on the detection of nucleocapsid [9]. The spike protein was not detectable. There can be several reasons for this, e.g. at this circumscribed location in the kidney it could indicate that complete viruses have not yet been synthesized there. The urine was negative at the time of the test. Whether viral replication had occurred in advance cannot be assessed. The detection of nucleocapsid in the kidney suggests viral replication. Whether this is ultimately directly causative of the renal injury or whether the injury results from an altered immune response in the setting of viral infection under high-level immunosuppressive therapy cannot be fully elucidated in the context of this case report.

In kidney transplant patients, a virus infection of the transplant should therefore also be considered in the event of a deteriorated graft function in the context of COVID-19. Before initiating therapy, the differential diagnoses of “rejection” and “virus infection” should be weighed against each other in an interdisciplinary team of nephrologists, infectious diseases specialists and pathologists.

## Conclusion

Lack of viral clearance can lead to complications of SARS-CoV-2 infection with atypical manifestations, such as infiltration of a renal graft, even late following initial infection in immunosuppressed individuals.

## Data Availability

All relevant data are shown in the manuscript.

## Abbreviations

eGFR: estimated glomerular filtration rate
FISH: Fluorescent in situ hybridization
iVIG: intravenous immunoglobulins
ELISpot: Enzyme Linked Immuno Spot Assay
SCr: Serum creatinine
IFTA: Interstitial fibrosis and tubules atrophy
